# Genomic characterization of *Listeria monocytogenes* recovered from dairy facilities in British Columbia, Canada from 2007 to 2017

**DOI:** 10.3389/fmicb.2024.1304734

**Published:** 2024-03-22

**Authors:** Stephanie R. B. Brown, Rebecca Bland, Lorraine McIntyre, Sion Shyng, Alexandra J. Weisberg, Elizabeth R. Riutta, Jeff H. Chang, Jovana Kovacevic

**Affiliations:** ^1^Food Innovation Center, Oregon State University, Portland, OR, United States; ^2^British Columbia Centre for Disease Control, Vancouver, BC, Canada; ^3^Department of Botany and Plant Pathology, Oregon State University, Corvallis, OR, United States

**Keywords:** *Listeria monocytogenes*, dairy environments, whole genome sequencing, genomic diversity, persistence

## Abstract

*Listeria monocytogenes* is a foodborne pathogen of concern in dairy processing facilities, with the potential to cause human illness and trigger regulatory actions if found in the product. Monitoring for *Listeria* spp. through environmental sampling is recommended to prevent establishment of these microorganisms in dairy processing environments, thereby reducing the risk of product contamination. To inform on *L. monocytogenes* diversity and transmission, we analyzed genome sequences of *L. monocytogenes* strains (*n* = 88) obtained through the British Columbia Dairy Inspection Program. Strains were recovered from five different dairy processing facilities over a 10 year period (2007–2017). Analysis of whole genome sequences (WGS) grouped the isolates into nine sequence types and 11 cgMLST types (CT). The majority of isolates (93%) belonged to lineage II. Within each CT, single nucleotide polymorphism (SNP) differences ranged from 0 to 237 between isolates. A highly similar (0–16 SNPs) cluster of over 60 isolates, collected over 9 years within one facility (#71), was identified suggesting a possible persistent population. Analyses of genome content revealed a low frequency of genes associated with stress tolerance, with the exception of widely disseminated cadmium resistance genes *cadA1* and *cadA2*. The distribution of virulence genes and mutations within internalin genes varied across the isolates and facilities. Further studies are needed to elucidate their phenotypic effect on pathogenicity and stress response. These findings demonstrate the diversity of *L. monocytogenes* isolates across dairy facilities in the same region. Findings also showed the utility of using WGS to discern potential persistence events within a single facility over time.

## 1 Introduction

Dairy product contamination with *Listeria monocytogenes* has led to numerous recalls and outbreaks worldwide (Desai et al., 2019). Since listeriosis, the disease caused by *L. monocytogenes*, is attributed to high rates of morbidity (>90%) and mortality (18–30%), it is imperative to minimize the pathogen’s entry into the food system (Silk et al., 2013; de Noordhout et al., 2014; Scobie et al., 2019). *L. monocytogenes* can be introduced into dairy foods and their associated environments through a variety of sources, including the farm/barn environments (Ho et al., 2007; Castro et al., 2018), udder surfaces (Castro et al., 2018), milking systems (Latorre et al., 2010; Castro et al., 2018), and milk (Fox et al., 2011). For dairy products requiring extensive handling, the production environment (e.g., drains and floors), equipment, and continuous use systems (e.g., brines) can also serve as contamination sources (Alessandria et al., 2010; Fox et al., 2011; Gaulin et al., 2012). Although pasteurization is an effective method to eliminate pathogens in these products, the presence of *L. monocytogenes* within the production environments presents a post-processing contamination risk to ready-to-eat (RTE) dairy products (Tompkin, 2002). To better understand the risk of product contamination within facilities, monitoring through environmental sampling is often used (Tompkin, 2002). This allows for the identification of contaminated areas and microbial niches that may be harboring *L. monocytogenes,* thereby providing opportunities for pathogen proliferation and continued introduction throughout the facility (Tompkin, 2002). Understanding contamination patterns helps prioritize and improve on existing cleaning and sanitation procedures and can indicate other systemic changes are warranted (e.g., review of employee practices and infrastructure modifications).

While prevalence within a facility is valuable information, leveraging these data with genetic information allows for a high level of discrimination between the recovered isolates. More specifically, these data can be used to further classify isolates into familial groupings (e.g., lineages and clonal complexes) that can be indicative of specific properties of these microorganisms (Orsi et al., 2011). At the broadest level, *L. monocytogenes* is grouped into four lineages, with most clinical and food-related isolates belonging to lineages I and II, respectively (Orsi et al., 2011). Although food-derived isolates are commonly associated with lineage II, sampling of dairy processing environments has shown the presence of both lineage I (Maury et al., 2019; Palacios-Gorba et al., 2021) and lineage II (Fox et al., 2011; Gaulin et al., 2012; Stessl et al., 2014) strains.

Genetic information gathered from sequencing data has also allowed for more specific classifications of *L. monocytogenes* based on conserved genes (Ragon et al., 2008). For example, allelic differences in seven housekeeping genes are used to assign isolates to a multi-locus sequence type (ST), while STs consisting of one or fewer allelic differences are grouped into clonal complexes (CCs) (Ragon et al., 2008). Notably, one CC (CC1; lineage I) has been strongly associated with dairy products in France (Maury et al., 2019) and ruminants (Dreyer et al., 2016; Palacios-Gorba et al., 2021).

Sequencing has also allowed for a better understanding of stress response mechanisms that may be indicative of persistence within the facility or survival in a food product (Belias et al., 2022). Specifically, single-nucleotide polymorphism (SNP) analysis has been used to show isolate similarities within and between facilities (Stasiewicz et al., 2015; Bland et al., 2021). This analysis has also been used to differentiate between sporadic and potentially persistent *L. monocytogenes* strains in retail delis (Stasiewicz et al., 2015), produce environments (Bland et al., 2021), patients, animals, and animal production environments (Camargo et al., 2019), meat and salmon raw materials, processed foods, and processing environments (Fagerlund et al., 2022), and dairy farms (Castro et al., 2021). Analyzing specific genes/genetic elements that are potentially or positively linked to stress response and/or virulence (e.g., antimicrobial tolerance genes and elements associated with invasion, pathogenicity islands or stress survival islets) may also provide insights into *L. monocytogenes* survival advantages and genetic diversity.

Due to decreasing costs and turn-around time for results, whole genome sequencing (WGS) is becoming a more viable option for use within multiple sectors of the food system (Brown et al., 2019). In recent years, North American agencies such as the Centers for Disease Control and Prevention, the Canadian Food Inspection Agency, and the Food and Drug Administration have transitioned to using WGS to identify and characterize pathogens of concern isolated from foods and food processing environments (Rantsiou et al., 2018; Brown et al., 2019). While zero tolerance for *L. monocytogenes* in all RTE foods is enforced in the U.S., in Canada, a tiered risk-based system is used for determining allowable limits (Health Canada, 2023). Specifically, zero tolerance is practiced for RTE foods that support the growth of *L. monocytogenes* and any RTE foods produced for vulnerable populations (Health Canada, 2023). For RTE products that do not support pathogen growth or have limited potential for growth (i.e., not exceeding 100 CFU/g), up to 100 CFU/g are allowed (Health Canada, 2023).

In 2002, two separate listeriosis outbreaks in British Columbia (BC), Canada were linked to the consumption of soft cheese, resulting in 134 illnesses (McIntyre et al., 2015). These outbreaks triggered the introduction of a voluntary monthly testing program of certain soft cheeses and collection of environmental samples by the industry and British Columbia Centre for Disease Control (BCCDC) inspectors (McIntyre et al., 2015). In this retrospective study, WGS was used to characterize *L. monocytogenes* isolates obtained through the BC Dairy Inspection Program over 10 years to better understand the diversity of *L. monocytogenes* isolates encountered in the dairy sector in BC.

## 2 Materials and methods

### 2.1 Whole genome sequencing and data assembly

*Listeria monocytogenes* isolates (*n* = 88) were previously isolated as part of provincial dairy inspection activities in BC, Canada, from 2007 to 2017. As part of inspectional activities, 87 finished product (food) and 195 environmental samples were collected from the five dairy facilities. Nine food product and 39 environmental samples were positive for *L. monocytogenes.* From these positive samples, 54 isolates from food samples and 34 isolates from environmental swab samples were selected for further analyses (Table 1). Isolates were stored at −80°C in tryptic soy broth (TSB; Neogen, Lansing, MI, United States) supplemented with 25% (w/v) glycerol. Prior to use, isolates were resuscitated by streaking onto tryptic soy agar (TSA; Neogen) supplemented with 0.6% yeast extract (YE; Fisher, Hampton, NH, United States), followed by 24 h incubation at 37°C. An isolated colony was transferred to 3 mL of TSB and incubated at 37°C for 18 h (TSB; Neogen). Genomic DNA was extracted using Qiagen DNeasy Blood and Tissue kits (Qiagen, Hilden, Germany) according to the manufacturer’s instructions for Gram-positive bacterial DNA. Quality and quantity of DNA was assessed using dsDNA broad range assay kit for DeNovix DS-11 Spectrophotometer/Fluorometer (DeNovix, Wilmington, DE, United States). DNA libraries were prepared at Oregon State University’s Center for Quantitative Life Sciences (Corvallis, OR, United States) using the PlexWell kit (seqWell, Beverly, MA, United States) according to the manufacturer’s instructions, followed by 2×150-bp paired-end sequencing using Illumina HiSeq (Illumina, San Diego, CA, United States). Resulting raw reads were quality checked with FastQC (v 0.11.9) and trimmed using Trimmomatic (v 0.39) (Bolger et al., 2014). Trimmed reads were *de novo* assembled using SPAdes (v 3.14.1) (Prjibelski et al., 2020) and optimized with Unicycler (v 0.4.8) (Wick et al., 2017). Resulting assembly files were assessed for quality (Supplementary Table S1) and annotated with Prokka (v 1.14.6) (Seemann, 2014).

**Table 1:** Number of samples and distribution of *L. monocytogenes* isolates, sequence types, clonal complexes, cgMLST types, *sigB* allelic profiles, and years of isolate recovery within each facility.

Facility/year	No. of samples (*n* = facility total)	No. of isolates	No. sequence types (ST)	No. clonal complexes (CC)	No. cgMLST types (CT)	No. *sigB* allelic types (AT)
Facility #14	*n* = 2					
2016	1	1	1	1	1	1
2017	1	1	1	1	1	1
Facility #71	*n* = 72					
2007	2	2	1	1	1	1
2009	3	9	2	2	2	2
2010	7	7	1	1	1	1
2012	12	45	2	2	2	2
2015	3	3	3	3	3	3
2016	6	6	2	2	2	2
Facility #106	*n* = 1					
2015	1	1	1	1	1	1
Facility #122	*n* = 12					
2017	12	12	1	1	1	1
Facility #131	*n* = 1					
2016	1	1	1	1	1	1

### 2.2 Multi-locus sequence typing, core genome MLST, and *sigB* allelic typing

Multi-locus sequence type (ST), clonal complex (CC), cgMLST, and *sigB* allelic profiles were assigned using the *Listeria* Pasteur database, BIGSdb-Lm (Moura et al., 2016). cgMLST was performed using a scheme consisting of 1,748 conserved core genes; assemblies were submitted to the BIGSdb-Lm to receive cgMLST type (CT) assignments.

### 2.3 Screening for virulence and stress response related genes

Isolates were screened for presence and absence of relevant virulence and stress response genetic markers according to Bland et al. (2021), with minor modifications. Briefly, the BIGSdb-Lm virulence scheme (92 loci) was used to assess virulence gene presence/absence. The presence of stress response genes, including antimicrobial resistance and tolerance genes (*tetR, tnpABC, qacH, qacC, emrE, emrC,* and *bcrABC*) and heavy metal genes (*cadA1, cadA2, cadA4, arsA1, arsA2, arsB1, arsB2, arsD1, arsD2, arsR1,* and *arsR2*) were screened using NCBI BLASTN, with a minimum nucleotide identity and alignment length coverage of 80%. Genetic mutations were identified by aligning genes of interest to a reference sequence using the MUSCLE algorithm in MEGAX (v 10.2.6) (Kumar et al., 2018). Accession numbers for reference strains used for genetic element screening and alignments are provided in the Supplementary Table S2.

### 2.4 Whole genome SNP analysis

Single nucleotide polymorphisms (SNPs) were assessed according to Weisberg et al. (2020), with slight modifications described by Bland et al. (2021). Briefly, the pairwise average nucleotide identity (ANI) among isolates and confirmation of species-level grouping (i.e., *L. monocytogenes*; >95% ANI) was performed using FastANI (v 1.1) (Jain et al., 2018). Raw reads were mapped to a representative reference sequence within the group (WRLP15) using BWA mem (v 0.7.17) (Li and Durbin, 2009). Alignments were annotated and sorted, and duplicate reads were identified using Picard tools (v 2.0.1). Graphtyper (v 2.6.2) was run with the default parameters (Eggertsson et al., 2017). The algorithm Graphtyper identifies sequence variants by aligning short read sequence data to a pangenome (Eggertsson et al., 2017). SNPs were filtered using vcffilter in vcflib (v 1.0.0) with the options -f “ABHet <0.0 | ABHet >0.33” -f “ABHom <0.0 | ABHom >0.97” -f “MaxAASR >0.4” -f “MQ > 30” (Garrison et al., 2021). SNP calls annotated as “FAIL” or “heterozygous” were filtered to “no-call.” Filtered SNP calls were converted to fasta format using BCFtools (v 1.3) (Li et al., 2009). The bitwise. Dist function within the R package poppr (v 2.9.2) was used to construct pairwise SNP distance tables from the fasta alignments (Kamvar et al., 2014). Poppr was then used to construct and visualize a minimum spanning network (MSN). The phylogenetic tree based on the SNP data was made using IQ-TREE version 1.6.12, with parameters -bb 1000 -alrt 1000 (Nguyen et al., 2015).

## 3 Results

### 3.1 General genome characteristics and isolate grouping

**Figure 1:**
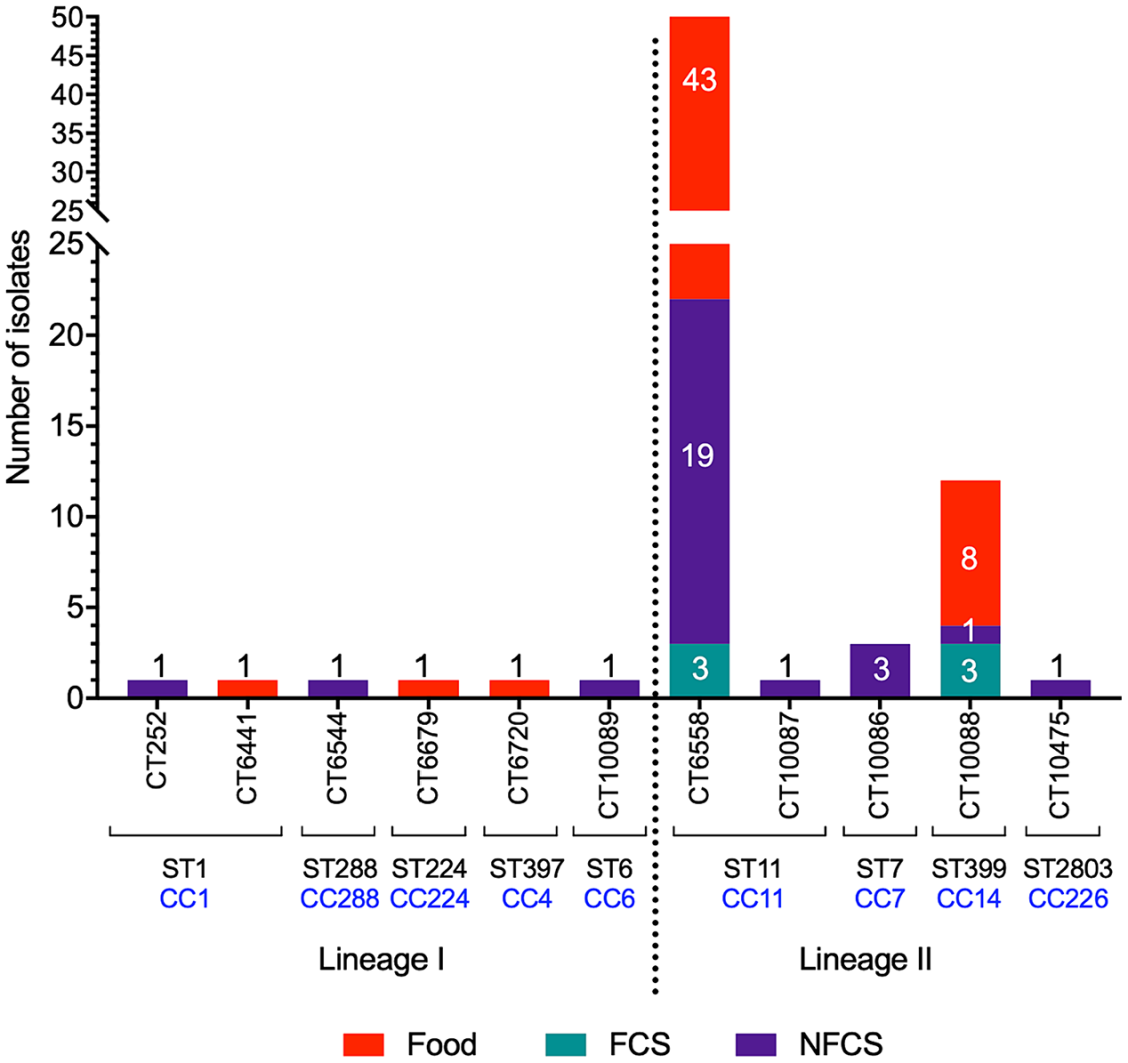
Sample origin and distribution of identified cgMLST types (CT), sequence types (ST), and clonal complexes (CC). Samples were collected from foods, food contact surfaces (FCS) or non-food contact surfaces (NFCS).

The majority of the isolates (*n* = 82) belonged to lineage II and only six isolates were lineage I. Lineage II isolates were recovered from foods (*n* = 51), food contact surfaces (FCS; *n* = 6) and non-food contact surfaces (NFCS; *n* = 25), whereas lineage I isolates were recovered from foods (*n* = 3) and NFCS (*n* = 3) (Figure 1). Classification information including isolate source, lineage, ST, CC, and CT is summarized in Tables 1, 2.

**Table 2:** *L. monocytogenes* isolate characterization and distribution of heavy metal and antimicrobial tolerance-related genes.

Facility/isolate ID	Year isolated	Source	Lineage	ST	CC	cgMLST	*bcrABC*	*cadA* genes
**Facility #14**								
WRLP94	2016	Environment	II	2803	226	10475	−	−
WRLP95	2017	Environment	II	11	11	10087	+	A1
**Facility #71**								
WRLP16-17	2007	Environment	II	11	11	6558	−	A1
DE25-1^a^, WRLP8-9	2009	Environment	II	7	7	10086	−	−
DE26-1^a^, DE27-1^a^	2009	Environment	II	11	11	6558	−	A1
WRLP11-12, WRLP14-15	2009	Environment	II	11	11	6558	−	A1
WRLP18-24	2010	Environment	II	11	11	6558	−	A1
WRLP26-68	2012	Food	II	11	11	6558	−	A1
WRLP69	2012	Environment	II	11	11	6558	−	A1
WRLP70	2012	Food	I	224	224	6679	−	−
WRLP71	2015	Environment	II	11	11	6558	−	A1
WRLP73	2015	Food	I	1	1	6441	−	−
WRLP74	2015	Food	I	397	4	6720	−	−
WRLP75-79	2016	Environment	II	11	11	6558	−	A1
WRLP80	2016	Environment	I	1	1	252	−	−
**Facility #106**								
WRLP81	2015	Environment	I	288	288	6544	−	A2
**Facility #122**								
WRLP82-83	2017	Environment	II	399	14	10088	−	−
WRLP84-87	2017	Food	II	399	14	10088	−	−
WRLP88-89	2017	Environment	II	399	14	10088	−	−
WRLP90-93	2017	Food	II	399	14	10088	−	−
**Facility #131**								
WRLP96	2016	Environment	I	6	6	10,089	−	−

a Isolates described by Kovacevic et al. (2012, 2013b).

There was a total of nine STs, belonging to nine CCs (Table 1). The majority (*n* = 66) of isolates were ST/CC11, followed by ST399/CC14 (*n* = 12), ST/CC7 (*n* = 3), ST/CC224 (*n* = 1), ST2803/CC226 (*n* = 1), ST/CC288 (*n* = 1) and the previously described hypervirulent ST/CC1 (*n* = 2; WRLP73 and WRLP80), ST397/CC4 (*n* = 1; WRLP74), and ST/CC6 (*n* = 1; WRLP96) (Maury et al., 2016) (Figure 1; Table 2). One ST was identified as novel: ST2803, belonging to CC226. Based on the cgMLST data, there were 11 CTs identified within this sample set, with CT6558 (ST/CC11) isolated at the highest frequency (*n* = 65), including isolates from food, FCS, and NFCS sources (Figure 1). Less commonly, isolates belonged to CT10088 (ST399, CC14; *n* = 12) and CT10086 (ST/CC7; *n* = 3), while only one isolate was seen in each CT252 (ST/CC1), CT6441 (ST/CC1), CT6544 (ST/CC288), CT6679 (ST/CC224), CT6720 (ST397, CC4), CT10087 (ST/CC11), CT10089 (ST/CC6), and CT10475 (ST2803, CC226) (Figure 1; Table 2). Based on *sigB* profiling, six different allelic types (ATs) were also identified, with the most common being AT 6 (*n* = 78), followed by AT 1 (*n* = 4), AT 2, and AT 4 (each *n* = 2), and single isolates belonging to each AT 3 and AT 13 (Table 1).

Isolate diversity varied between facilities. Two facilities (#106 and #131) each had only one isolate recovered, limiting analyses within the facility (Table 1). Facility #14 had two isolates recovered, in different years, both with distinct ST, CC, CT, and *sigB* AT profiles (Table 1). In contrast, 12 isolates recovered in facility #122 in the same year all had the same ST, CC, CT, and *sigB* AT profiles (Table 1). The majority of the analyzed isolates came from facility #71, recovered from 2007 to 2016. This facility also had the highest isolate diversity, with 72 isolates belonging to five ST, CC, and *sigB* AT profiles and six CTs (Table 1). Novel CTs were recovered from four of the five facilities sampled. Specifically, five novel CTs were discovered, including two novel CTs recovered from facility #14 (CT10475 and CT10087), and one novel CT isolated from each of these facilities #71 (CT10086), #122 (CT10088), and #131 (CT10089) (Table 2).

### 3.2 SNP analysis

**Figure 2:**
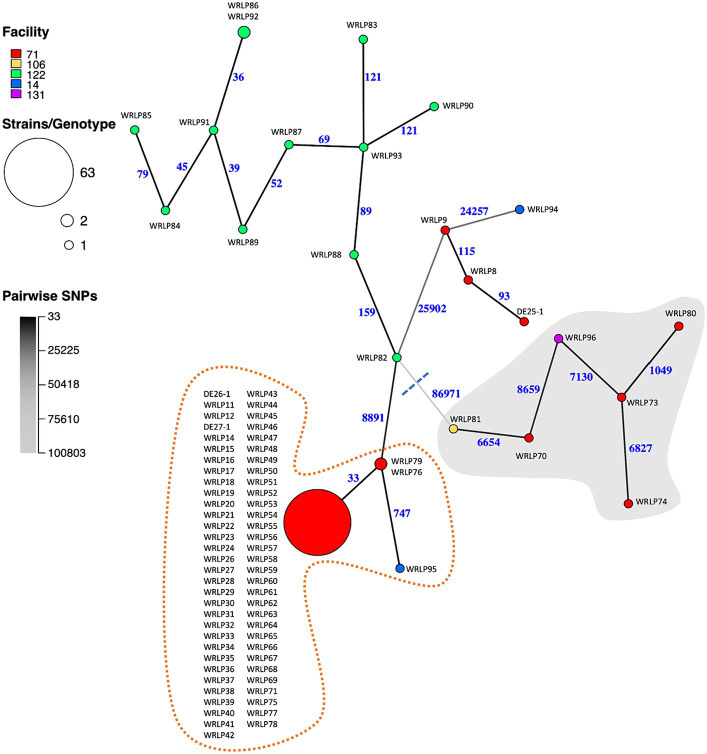
Minimum spanning network (MSN) of whole genome SNP differences identified among the 88 *Listeria monocytogenes* isolates collected from BC dairy facilities. Nodes represent genotypes [defined based on a threshold of <16 pairwise SNP differences (Weisberg et al., 2021; Iruegas-Bocardo et al., 2022)]. Node sizes are proportional to the number of isolates belonging to the genotype. Node colors indicate facility from which the isolates were collected. Branch color and numbers indicate the number of pairwise SNP differences between genotypes (i.e., darker colors indicate fewer differences). Gray background shows isolates belonging to lineage I, whereas the white background shows isolates belonging to lineage II. Dotted orange line highlights isolates belonging to ST11.

The pairwise SNP differences among the isolates ranged from 0 to 103,956 (Supplementary Figure S1). As expected from the genetic distance between lineages, the largest SNP difference was seen between a lineage I strain, WRLP74 (ST397/CC4), and a lineage II strain, WRLP94 (ST2803/CC226), recovered from facilities #71 and #14, respectively (Figure 2; Supplementary Figure S1). For the CTs containing more than one isolate, pairwise SNP differences varied from 0–33 (CT6558) and 92–139 (CT10086) to 9–237 (CT10088) (Supplementary Figure S1). We next mapped facility information onto the MSN to infer transmission patterns and potential for persistent populations. The most remarkable pattern was observed for facility #71. It had a diversity of isolates from both lineages I and II, consistent with multiple, separate introductions. However, the majority of isolates (*n* = 63) grouped into a single genotype (ST/CC11; Figure 2). Given that members of this genotype were collected throughout the 9 years of sampling (Table 2), this pattern is less likely explained by introduction of a large population and more consistent with the presence of a persistent population. Facility #122 also had numerous isolates, and all but one pair were categorized into unique genotypes, with SNP differences ranging from 36 to 159 (Figure 2). When compared to isolates from other facilities, isolates from facility #122 had a minimum of 9,314 and a maximum of 100,056 SNP differences (Figure 2; Supplementary Figure S1). Facility #14 had two isolates (WRLP94 and WRLP95; ST2803/CC226 and ST/CC11, respectively) recovered, and they had 31,881 pairwise SNP differences (Supplementary Figure S1). Though ST11 was observed in more than one facility, the isolate collected from facility #14 (WRLP95) and a cluster of 65 ST11 isolates from facility #71 differed by 744–778 pairwise SNP differences (Figure 2; Supplementary Figure S1).

### 3.3 Genetic elements associated with stress tolerance

**Figure 3:**
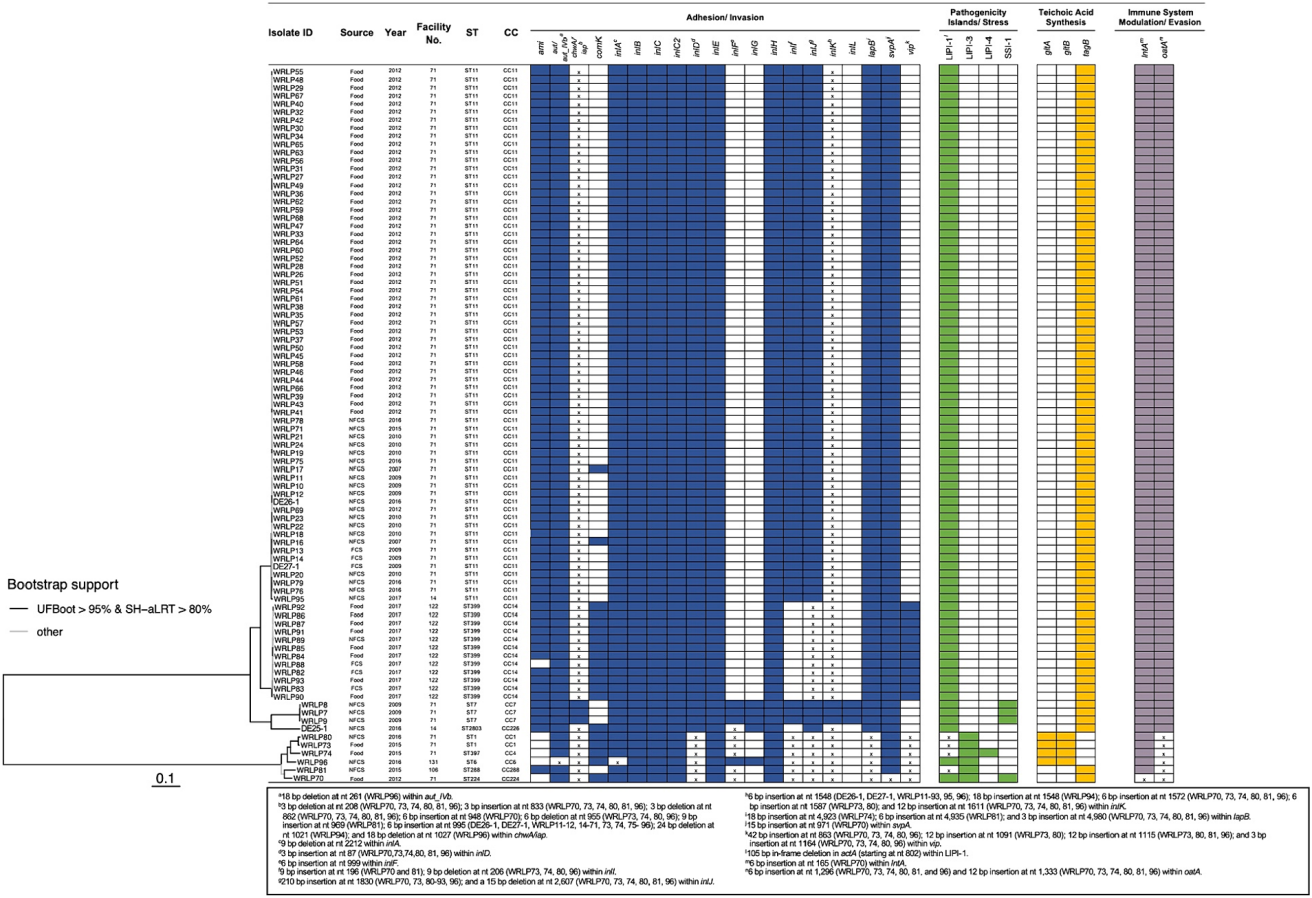
Presence of genetic elements associated with virulence and stress response in *L. monocytogenes* isolates recovered from dairy facilities. The phylogenetic tree, based on SNP data, was obtained using IQ-TREE version 1.6.12 with the parameters -bb 1000 and -alrt 1000. Shaded boxes indicate the presence of the genetic element; white boxes indicate the absence of the genetic element; x indicates insertion(s) or deletion(s) identified within the respective gene or genetic element. No premature stop codons (PMSCs) were identified in any genes containing insertions or deletions. Genetic elements with different colored boxes have differing genetic functions.

A low frequency of genes and genetic elements associated with stress tolerance and antimicrobial resistance (AMR) was seen among the isolates (Table 2; Figure 3). Importantly, genes and genetic elements present in more than one isolate were also seen in other isolates belonging to the same ST/CC. The *bcrABC* cassette, associated with tolerance to quaternary ammonium compounds, was identified in the genome sequence of only one isolate (WRLP95; ST/CC11) (Table 2). Other genes associated with AMR and stress tolerance (e.g., *tetR, qacC, qacH, tnpABC,* and *emrC*) were not identified in any genome sequences examined. Also, none of the genomes possessed the *Listeria* genomic island (LGI) 1, *cadA4, emrE,* or the arsenic resistance gene cassette located in the LGI2. However, genetic determinants associated with the tolerance to the heavy metal cadmium (*cadA1* or *cadA2*) were detected in the genome sequences of isolates recovered from three facilities (#14, #71, and #106) (Table 2). Specifically, *cadA1* was found in 75% (66/88) of isolates, whereas the *cadA2* gene was only identified in WRLP81 (ST/CC288). Notably, the *cadA1* gene was identified in 65 (91%) isolates from facility #71, and one of two isolates recovered from facility #14 (all ST/CC11).

The stress survival islet (SSI-1) was only identified in isolates within facility #71, including isolates recovered in 2009 (DE25-1, WRLP8, and WRLP9; ST/CC7) and 2012 (WRLP70; ST/CC224) (Figure 3). When comparing the SSI-1 genes of these four isolates, three isolates recovered in 2009 (DE25-1, WRLP8, and WRLP9; ST/CC7) had no SNP differences across all five genes in SSI-1. The isolate that was recovered in 2012, WRLP70 (ST/CC224), had two SNP differences in *lmo0447*, three SNP differences in *lmo0445*, four SNP differences in *lmo0446* and *lmo0448*, and 11 SNP differences in *lmo0444* gene as compared to the SSI-1 genes from the 2009 isolates.

### 3.4 Presence of virulence genes

A select number (*n* = 92) of genes and genetic elements known to confer virulence were investigated in all isolates, with the results indicating a high level of variability in their presence/absence and sequence similarity (Figure 3). Overall, there were 50 virulence genes present in all isolates, 16 virulence genes absent in all isolates, and 26 genes present within a subset of the isolates. Premature stop codons (PMSCs) were not observed in any of the *inlA* and *inlB* genes. However, a 9-bp internal deletion within *inlA* was found in WRLP96 (nt 2212 to 2220; ST/CC6). Other internalin genes were observed in all or a subset of isolates. Specifically, *inlC*, *inlC2*, *inlD*, *inlE*, *inlH*, *inlJ*, and *inlK* were present in all 88 tested isolates, whereas the presence of other internalins was dependent on lineage and clonal complex. Specifically, *inlF* was present in all lineage I isolates and 4/82 (5%) lineage II isolates (ST/CC7: DE25-1, WRLP8, and WRLP9; and ST2803/CC226: WRLP94). With the exception of WRLP96 (ST/CC6), all lineage I isolates, and one lineage II isolate (WRLP94) had a 6-bp insertion at nt 999 within *inlF*. Similarly, *inlG* was found in five isolates, belonging to ST/CC7, ST2803/CC226, and ST/CC6. Three isolates from facility #71, belonging to ST/CC7, harbored *inlL*, whereas *inlI* was present in most of the tested isolates, with the exception of ST399/CC14 (WRLP82-93) and ST2803/CC226 (WRLP94) isolates. For all lineage I isolates, indel mutations were present in the *inlI* gene. Similarly, a 3-bp insertion (nt 87) was present in the *inlD* gene of all lineage I isolates. Several isolates contained a 210-bp insertion (at nt 1830; *n* = 18) and some isolates exhibited a 15 bp deletion (at nt 2607; *n* = 6) in *inlJ*; whereas the majority of isolates (*n* = 85) possessed insertions within *inlK* when aligned to a reference strain (EGDe; accession no. NC_003210) (Supplementary Table S2).

The presence of other virulence genes varied depending on lineage, CC, and facility of isolate origin (Figure 3). The adhesion gene *ami* was absent in all lineage I isolates from facility #71, one isolate from facility #122 (WRLP88; lineage II; ST399/CC14), and one isolate from facility #131 (WRLP96; lineage I; ST/CC6). Conversely, *vip* was absent from most of the facility #71 isolates, with the exception of the four lineage I isolates (WRLP70, WRLP73, WRLP74, and WRLP80), which all had nucleotide insertions within the gene. The *vip* gene was also found in isolates recovered from facility #106 (WRLP81; lineage I; ST/CC288), facility #122 (WRLP82-WRLP93; lineage II; ST399/CC14) and facility #131 (WRLP96; lineage I; ST/CC6). A gene associated with vacuolar escape, *comK*, was present in all lineage I isolates and two lineage II isolates from facility #71, all isolates from facility #122, and one isolate in each facility #14 and #131. Genes *lapB, lntA, oatA,* and *svpA* were present in all isolates. However, insertions were identified in the *lapB* and *oatA* genes of all lineage I isolates, and 6 bp and 15 bp insertions were identified in the *lntA* and *svpA* genes, respectively, within WRLP70 (ST/CC224). For the teichoic acid synthesis genes, all isolates either contained *gltA* and *gltB* or *tagB.* Specifically, *gltA* and *gltB* were present in 4/6 lineage I isolates (WRLP73, WRLP74, WRLP80, and WRLP96), whereas *tagB* was present in all other isolates. The invasion gene *aut,* or its allelic form *aut_IVb,* was present in all isolates within this dataset, with a 18 bp deletion noted at nt 261 in WRLP96 (ST/CC6). For the *iap* (also known as *chwA*) gene, indel mutations were present in all isolates except those belonging to ST/CC7 (DE25-1, WRLP8, and WRLP9).

For the *Listeria* pathogenicity islands, all six LIPI-1 genes (*prfA, plcA, hlyA, mpl, actA,* and *plcB*) were present in all the *L. monocytogenes* isolates examined (Figure 3). However, a 105 bp internal deletion in *actA* was observed in the isolates belonging to ST/CC1, ST397/CC4, and ST/CC288 (WRLP73 and WRLP80, WRLP74, and WRLP81, respectively) at nt position 793. LIPI-2 was not detected in any of the isolates, while LIPI-3 and LIPI-4 were only found among lineage I isolates. All six lineage I isolates possessed LIPI-3 genes (*llsG, llsH, llsX, llsB, llsY, llsD, llsP,* and *llsA*), while WRLP74, belonging to ST397/CC4, was the only isolate possessing LIPI-4.

## 4 Discussion

Understanding the population dynamics and diversity of *L. monocytogenes* found in frequently consumed food commodities within a region is essential for effective public health risk management, disease surveillance and prevention of food contamination. The *L. monocytogenes* isolate set recovered through the BC Dairy Inspection Program provided the opportunity to use whole genome sequencing information to investigate population diversity of isolates recovered in dairy facilities located within the same geographic region over a long period of time, improving our understanding of *L. monocytogenes* genotypes within and across facilities, their virulence potential, and contamination and persistence events.

Although some of the dairy isolates characterized in this study belonged to hypervirulent CCs within lineage I and possessed unique virulence elements (Table 2), the majority (93%) of isolates belonged to lineage II. This is in contrast to findings from a large longitudinal study from France, which reported that lineage I strains were strongly associated with dairy products (Maury et al., 2019), and a large-scale longitudinal study of dairy farms in Spain, where lineage I accounted for 69% of isolates (Palacios-Gorba et al., 2021). While some studies have suggested that lineage II isolates are more often associated with foods and environments than clinical cases (Orsi et al., 2011; Maury et al., 2016), in Canada lineage II isolates, especially strains belonging to CC8, have been predominantly associated with outbreaks and sporadic cases of listeriosis for more than two decades (Knabel et al., 2012). Notably, none of the isolates examined here belonged to CC8. These findings suggest that drivers behind ecology and genomic characteristics of *L. monocytogenes* in dairy ruminants and farms and dairy facilities may be diverse, especially in different geographic locations.

When comparing isolates by CCs across the five dairy processing facilities, only ST/CC11 was found in more than one facility (facilities #71 and #14) (Table 2). This is not surprising, as previous studies in cheese processing (Fox et al., 2011) and milk-associated environments (Ebner et al., 2015) reported that isolates from different facilities tend to have more genomic differences compared to isolates recovered within the same facility. Notably, in our study two facilities had isolates belonging to the same CC recovered during different sampling events; ST399/CC14 in facility #122 from samples collected 10 days apart, and ST/CC11 in facility #71 from samples collected 9 years apart (Table 2). Unlike other reports where one CC was strongly associated with dairy products (Maury et al., 2019) and ruminants (Dreyer et al., 2016; Palacios-Gorba et al., 2021), we saw a considerable CC diversity in dairy environments, even from facilities located within the same geographic area. These findings suggest that contamination events are likely driven by different activities conducted within each facility, and contamination events are highly dependent on the conditions practiced within a facility at the time of sampling. In some facilities contamination is likely to be transient with occasional positives and different CCs, such as in facilities #14, #106, and #131 (Table 1). It is also possible that in some facilities, a contamination event can lead to dispersal of *L. monocytogenes* of the same CC within food and the environment; however, it is not necessarily a persistent contamination, but rather a short-term but widely disseminated contamination event, a potential scenario in facility #122. In more problematic cases, the contamination can be due to a harborage site and/or persistent CC genotypes, and/or a more systematic failure to control *L. monocytogenes*, leading to recurrent positives and dispersal across the facility over a prolonged period of time, such as in facility #71.

These findings highlight the importance of using whole genome sequences and more discriminatory analyses, such as SNPs, to better understand potential contamination and persistence events and genetic diversity of *L. monocytogenes* contaminants within a facility (Figure 2). Within the BC isolate set, the group of 12 ST399/CC14/CT10088 isolates within facility #122 were clearly differentiated based on SNP differences (39–159 SNPs), suggesting highly similar but unique isolates. These genetic similarities could be the result of the same original contamination source, or less likely, they could be from separate contamination events. The group of 65 CT6558 isolates (ST/CC11), however, was less clearly separated, with isolates being indistinguishable or closely related based on SNP differences (0–33) (Figure 2). This highly similar group of isolates from the same facility (#71) suggests a potential harborage site and/or dispersal throughout the facility. Since these isolates were collected over multiple years (2007–2016), this further suggests persistence (Kwong et al., 2016) of a specific genotype occurring within facility #71.

While persistence has been speculated to be associated with stress response genes, such as genes associated with antimicrobial resistance and tolerance (e.g., *bcrABC*, *qacC*, or *emrE*) (Kovacevic et al., 2016; Cooper et al., 2021), isolates recovered from BC facilities were largely devoid of these genes (Table 2). One exception was isolate WRLP95 (ST/CC11), from facility #14, which harbored the *bcrABC* resistance cassette associated with tolerance toward quaternary ammonium compounds (Dutta et al., 2013). Similar findings of low prevalence of antimicrobial tolerance genes have been reported in a number of other studies that examined isolates from dairy processing and associated environments (Ebner et al., 2015; Palacios-Gorba et al., 2021; Palaiodimou et al., 2021), while a French study that included food and clinical isolates collected over 12 consecutive years reported a much higher prevalence of these genetic markers (32%) (Maury et al., 2019). In the current study, one potential explanation for this observation is the overrepresentation of isolates collected from facility #71 compared to the number of samples collected from other facilities. Most of these isolates are likely from a persistence event, based on SNP analysis, and none of these isolates contained these genes of interest.

Genetic determinants for heavy metal resistance, however, were widely observed in BC isolates (Table 2). Similar results have been reported in other studies that examined dairy isolates, with resistance to heavy metal cadmium commonly seen (Almeida et al., 2013; Castro et al., 2021). While the role of heavy metal resistance genes in persistence events remains unclear, the prevalence of genes conferring cadmium tolerance suggests that it could be assisting in overall stress survival in these types of environments.

Similarly, the presence of stress survival islet 1 (SSI-1), consisting of five genes that assist in *L. monocytogenes* survival under stress conditions, especially in acidic and elevated salt environments (Ryan et al., 2010), has been suggested to increase survival of *L. monocytogenes* in food industry environments. Since organic acids can be naturally found or added to fermented dairy products, it is likely that this islet is beneficial for *L. monocytogenes* survival in dairy facilities. However, SSI-1 was not highly prevalent among BC isolates, with only four isolates from the same facility (#71) possessing full length SSI-1 (Figure 3). Of particular interest, three of these isolates belonged to CC7, which has previously been shown to have a significantly higher tolerance to lactic acid compared to 14 other CCs studied by Myintzaw et al. (2022). Other studies have also proposed the presence of SSI-1 aiding in biofilm formation and increased adherence (Keeney et al., 2018), and impacting quaternary ammonium compound tolerance (Bland et al., 2021), giving the isolates increased survival advantage in a facility. With many of the isolates not possessing SSI-1 and being repeatedly recovered from facility #71 over the years, it is unclear what role and if any survival advantage was afforded to the four isolates that possessed SSI-1 in the studied isolate set.

Studies have also suggested that in some *L. monocytogenes* strains the *comK* gene and prophage gene insertions can be used as markers to differentiate outbreak strains (Chen and Knabel, 2008), and that under specific conditions these genes may allow *L. monocytogenes* to rapidly adapt to different food processing facilities and foods (Verghese et al., 2011). *comK* regulates the DNA uptake competence system in *L. monocytogenes,* which aids in bacterial escape from host phagosomes (Rabinovich et al., 2012). Prophage insertion in this gene renders it non-functional, requiring prophage excision prior to gene expression (Rabinovich et al., 2012; Johansson and Freitag, 2019). The prophage is excised during intracellular growth, thereby acting as a genetic switch to modulate virulence (Rabinovich et al., 2012; Johansson and Freitag, 2019). In the present study intact *comK* was seen in 23% (20/88) of isolates, including all isolates from facilities #122 and #131, 8% of isolates from facility #71, and in one isolate from facility #14 (Figure 3). Higher rates of intact *comK* genes have been reported in studies examining isolates from non-dairy sources, including 100% (28/28) of *L. monocytogenes* isolates from fish and fish processing environments in Poland (Wieczorek et al., 2020), 47% (47/100) of isolates collected from three Irish meat and vegetable facilities over a four-year sampling period (Hurley et al., 2019), and 40% (21/52) of isolates recovered from multiple food sources over an 18-year period in Australia (Gray et al., 2021). At present, it is not clear whether isolates with intact *comK* have virulence or other survival advantage over those isolates that require prophage excision.

When classical markers associated with adhesion and invasion properties, such as internalins *inlA* and *inlB*, were examined, there were no PMSCs observed to suggest reduced invasion capacity (Nightingale et al., 2008; Kovacevic et al., 2013a; Chalenko et al., 2019). With the exception of one ST/CC6 strain possessing 9-bp deletion in *inlA* (WRLP96), which was previously reported in several studies and not associated with decreased invasiveness (Kovacevic et al., 2013a; Kanki et al., 2015), all strains had intact *inlA* and *inlB* genes, indicative of virulence potential (Figure 3). Notably, none of the lineage II strains had *inlA* PMSCs, which have previously been suggested to occur in as high as 30% of lineage II isolates originating from food and food processing environments (Van Stelten et al., 2010; Orsi et al., 2011; Kovacevic et al., 2013a).

Although the role of other internalins in virulence has not been clearly established, considering the presence, absence and mutations in these genes may offer a greater level of discrimination among *L. monocytogenes* strains. This has been suggested to be helpful in partial profiling to discriminate between suspected transient and persistent contamination events within a facility (Jia et al., 2007). Genotyping of 31 clinical and food chain isolates from Ireland found *inlA, inlB, inlC, inlC2, inlD, inlH, inlE, inlI*, and *inlJ* in all tested isolates, whereas *inlF* was present in 28/31 isolates (Hilliard et al., 2018). Similar to our study, a 9-bp deletion in *inlA* in three CC6 isolates was reported (Hilliard et al., 2018) (Figure 3). The overall prevalence of these internalin genes is in line with the results of this study, suggesting that *inlF* is sporadically found across the species. Sporadic findings of *inlF* were also noted by Papić et al. (2019); specifically, *inlF* in CC11 (ST1279) isolates was suggested to contribute to the differences in cattle disease manifestations (Papić et al., 2019).

In the set of isolates analyzed in our study, mutations were identified in *inlD, inlF, inlI, inlJ,* and *inlK* (Figure 3). Most of these mutations were present in the lineage I isolates, except for *inlJ* mutations, which were also found in lineage II isolates from facility #122. In contrast to our findings, others have reported intact *inlJ* genes in *L. monocytogenes* isolates recovered from raw milk (Jamali and Radmehr, 2013). The mutations in *inlK* were found in isolates from every sampled BC facility and only absent from ST/CC7 isolates from facility #71. To the best of our knowledge, the roles of these mutations have not been established. However, mutants lacking the *inlG*, *inlH*, and/or *inlE* genes have been reported to have increased internalization in HBMEC and Caco-2 cell lines (Bergmann et al., 2002). While the genotypic lack of *inlG* in most of the isolates from this set suggests a phenotypic increase in virulence potential among these isolates, phenotypic assays are necessary to assess the effect of the absence of this gene. Based on previous studies showing that the presence/absence and mutations within internalin genes plays a role in virulence (Kovacevic et al., 2013a), it would be of interest to further explore the virulence potential of the isolates within this study.

Similar to internalins, *Listeria* pathogenicity islands (LIPIs) have been traditionally used to further screen *L. monocytogenes* virulence potential (Disson et al., 2021). Four major LIPIs have been described in *L. monocytogenes* (LIPI-1 to -4) (Disson et al., 2021; Lee et al., 2023). The main island responsible for aiding in the escape of *L. monocytogenes* from the host cell and evasion of the host immune system, LIPI-1, is widely conserved across the species (Disson et al., 2021). As expected, it was present in all BC isolates that we tested; however, mutations were identified within the *actA* gene of four lineage I isolates (Figure 3). ActA plays several important roles in pathogenicity, including vacuole escape and actin polymerization, which drives actin-based motility for *L. monocytogenes* cellular spread among host cells (Vazquez-Boland et al., 2001; Travier and Lecuit, 2014). For the four isolates with a 105 bp deletion within the *actA* gene, the deletion was seen in the first proline-rich repeat (PRR) region (Smith et al., 1996). The same deletion was noted in *L. monocytogenes* isolates collected from a mushroom facility in the Netherlands (Lake et al., 2021). Similar sized deletions have been found by other groups within the PRR region but the specific location was not noted (Chen et al., 2009; Bland et al., 2021) or the deletion was characterized in another area of the PRR region of *actA* (Jiang et al., 2006; Holch et al., 2010). More studies are needed to fully understand the impact of these *actA* PRR region deletions on *L. monocytogenes*, as some phenotypic studies have shown no impact on virulence (Chen et al., 2009; Holch et al., 2010), while others have reported potentially reduced virulence in mice and chicken embryos (Jiang et al., 2006).

When examining other pathogenicity islands, no isolates possessed LIPI-2. LIPI-3 was present in six isolates all belonging to lineage I, whereas LIPI-4 was seen in one isolate only, also belonging to lineage I (Figure 3). LIPI-3 genes encode for the hemolytic, cytotoxic virulence factor listeriolysin S (Cotter et al., 2008), while LIPI-4 genes encode for a cellobiose-type phosphotransferase system and are typically found in CC4 isolates, believed to contribute to their hypervirulent phenotype (Maury et al., 2016). Stronger association with lineage I strains and LIPI-3 has been reported in some studies (Cotter et al., 2008; Zakrzewski et al., 2023), though it can also be found in lineage II strains (Zakrzewski et al., 2023), while LIPI-4 has been reported in both lineage I and II strains, frequently among clinical strains isolated from infections of the central nervous system and placenta (Maury et al., 2016). Notably, LIPI-3 and LIPI-4 have also been seen in non-pathogenic species of *Listeria*, with full-length LIPI-4 reported in all of 36 *L. innocua* strains examined by Lee et al. (2023) with ~85% similarity to LIPI-4 in *L. monocytogenes*, and truncated versions of LIPI-3 seen in *L. innocua* and *L. seeligeri* strains. Presently, it is not clear what survival advantage the islands afford to nonpathogenic species, though evidence suggests different *Listeria* spp., pathogenic and nonpathogenic ones, acquire these islands via horizontal gene transfer likely as a result of different selection pressures (Lee et al., 2023).

Other virulence factors, such as *vip* and *ami*, have been explored in some studies as additional markers of virulence (Milohanic et al., 2001; Cabanes et al., 2005). *vip* aids in *L. monocytogenes* entry into select types of host cells (Cabanes et al., 2005). Its importance has been suggested in the last few years due to its presence in several outbreak strains (Rychli et al., 2014; Luth et al., 2020). The differences identified in outbreak isolates coupled with *vip*’s absence in other *Listeria* spp. (Cabanes et al., 2005) make this gene a potential candidate for future partial profiling applications; however, more research is needed to better understand the distribution of *vip* across isolates on a global scale. In this study, the *vip* gene was detected in all lineage I isolates, albeit with various insertions at different nucleotide positions within the gene, and in all isolates recovered from facility #122 belonging to lineage II (Figure 3). These insertions were not associated with PMSCs in this gene. In contrast, Steingolde et al. (2021) did not detect *vip* in lineage I isolates (CC2, CC4, and CC6; n = 3), while there have been several reports of it being present in lineage II *L. monocytogenes* isolates recovered from different sources over multiple sampling years (Steingolde et al., 2021; Schiavano et al., 2022). At the CC/ST level, our findings were similar to those reported by Steingolde et al. (2021) for CC7, CC226, and CC14 isolates from cattle abortion cases, where *vip* was absent in all CC7 and CC226 isolates and present in CC14 isolates; however, data for CC11 differed. Based on the variability of data, at present there is no evidence of particular ST or CCs being a reliable indicator of *vip* presence or absence, and to what extent *vip* contributes to *L. monocytogenes* virulence.

Similarly, the role of *ami* in *L. monocytogenes* virulence is not clear. While this gene has been found in other, nonpathogenic *Listeria* spp. including *L. innocua* and *L. welshimeri* (Hain et al., 2006), the Ami proteins in these nonpathogenic species are typically truncated compared to *L. monocytogenes* (Hain et al., 2006). Almost all dairy isolates sequenced in this study contained *ami* except for several lineage I isolates from facilities #71 (*n* = 4) and #131 (*n* = 1), and one lineage II isolate from facility #122 (WRLP88; ST399/CC14) (Figure 3). When looking at *L. monocytogenes* isolates from different global outbreaks, there are examples of isolates that had intact *ami* genes, truncated *ami* genes, and an isolate without the gene (Wagner et al., 2021). Truncated *ami* was also identified in some ST14 and ST121 *L. monocytogenes* isolates considered to be potentially persistent from a rabbit meat processing plant and 23 publicly available *L. monocytogenes* genomes (Palma et al., 2017). Others have found *ami* present in all isolates (*n* = 450) characterized from clinical and food sources (Jacquet et al., 2002). However, gene presence does not seem to guarantee protein functionality, with protein production reported as absent in some serotype 4b isolates (Jacquet et al., 2002). While many isolates from BC had *ami*, additional work is needed to determine if the Ami protein is expressed, and to what extent this protein affects virulence potential.

## 5 Conclusion

By evaluating *L. monocytogenes* isolates obtained from dairy processing facilities across BC, insights were gained into the diversity of isolates within and across these facilities. Even though samples were collected based on a regulatory sampling assignment (i.e., not a predetermined experimental design), and not all samples yielded *L. monocytogenes* positive results, analyzing the collected isolates and conditions in the facility at the time of sampling can be useful in understanding population dynamics within facilities producing similar commodities in the region as well as in providing information about potential strain persistence within dairy environments. Outside of regulatory activities, the sporadic finding of positive samples in dairy environments highlights the importance of routine sampling with the intent to find the pathogen as part of an environmental monitoring program or other sampling plan within facilities to help reduce the risk of product contamination and illness risk to the consumer. Whole genome sequencing helped identify isolate similarities and differences, and several virulence and stress tolerance genetic markers that could be used by facilities to differentiate *L. monocytogenes* within their food production environment(s). These data also highlight that different lineages, clonal complexes and sequence types are dominant in dairy environments in different regions, especially between North American and European studies, cautioning researchers and authorities not to rely on studies from limited regions, whether longitudinal or short term, in risk assessment studies and models.

## Data availability statement

The datasets presented in this study can be found in online repositories. The names of the repository/repositories and accession number(s) can be found at: Bioproject accession PRJNA998448.

## Author contributions

SB: Data curation, Formal analysis, Methodology, Visualization, Writing – original draft, Writing – review & editing. RB: Data curation, Formal analysis, Visualization, Writing – review & editing. LM: Conceptualization, Writing – review & editing. SS: Conceptualization, Writing – review & editing. AW: Formal analysis, Methodology, Supervision, Validation, Visualization, Writing – review & editing. ER: Data curation, Writing – review & editing. JC: Supervision, Validation, Writing – review & editing. JK: Conceptualization, Formal analysis, Funding acquisition, Methodology, Project administration, Supervision, Visualization, Writing – original draft, Writing – review & editing.
