# Supplementary material for: Genomic characterization of Listeria monocytogenes recovered from dairy facilities in British Columbia, Canada from 2007 to 2017
Source: Front Microbiol. 2024 Mar 22;15:1304734. doi: 10.3389/fmicb.2024.1304734 (PMC10995413; doi:10.3389/fmicb.2024.1304734)
Supplement: Supplementary file 1 [file Table_1.DOCX]

Supplementary Material

Genomic characterization of *Listeria monocytogenes* recovered from five dairy facilities in British Columbia, Canada from 2007 to 2017

Stephanie R. B. Brown^1^, Rebecca Bland^1^, Lorraine McIntyre^2^, Sion Shyng^2^, Alexandra J. Weisberg^3^, Elizabeth R. Riutta^3^, Jeff H. Chang^3^, and Jovana Kovacevic^1*^

*** Correspondence:**Jovana Kovacevic

Email: [jovana.kovacevic@oregonstate.edu](mailto:jovana.kovacevic@oregonstate.edu)

**Table S1** | Quality of *L. monocytogenes* assemblies after SPAdes *de novo* assembly and Unicycler optimization.

| **Strain ID** | **Facility** | **Contigs No.** | **Length (bp)** | **N_50_** |
| --- | --- | --- | --- | --- |
| WRLP94 | 14 | 17 | 2871380 | 1451663 |
| WRLP95 | 14 | 36 | 3099846 | 459593 |
| DE25-1 | 71 | 31 | 2941926 | 376367 |
| DE26-1 | 71 | 47 | 3046937 | 508930 |
| DE27-1 | 71 | 58 | 3049053 | 508930 |
| WRLP8 | 71 | 28 | 2942232 | 376381 |
| WRLP9 | 71 | 31 | 2941927 | 376367 |
| WRLP11 | 71 | 49 | 3046532 | 508930 |
| WRLP12 | 71 | 47 | 3046938 | 508930 |
| WRLP14 | 71 | 56 | 3049462 | 508930 |
| WRLP15 | 71 | 56 | 3049463 | 508931 |
| WRLP16 | 71 | 22 | 3020345 | 539558 |
| WRLP17 | 71 | 22 | 3020344 | 539558 |
| WRLP18 | 71 | 55 | 3047109 | 508930 |
| WRLP19 | 71 | 56 | 3049462 | 508930 |
| WRLP20 | 71 | 60 | 3048724 | 508930 |
| WRLP21 | 71 | 55 | 3047693 | 508930 |
| WRLP22 | 71 | 41 | 3044425 | 508930 |
| WRLP23 | 71 | 37 | 3041518 | 508930 |
| WRLP24 | 71 | 52 | 3047668 | 508930 |
| WRLP26 | 71 | 61 | 3049790 | 508930 |
| WRLP27 | 71 | 61 | 3049730 | 508930 |
| WRLP28 | 71 | 59 | 3050198 | 508930 |
| WRLP29 | 71 | 59 | 3050402 | 508930 |
| WRLP30 | 71 | 59 | 3050198 | 508930 |
| WRLP31 | 71 | 63 | 3049460 | 508930 |
| WRLP32 | 71 | 62 | 3050062 | 508930 |
| WRLP33 | 71 | 61 | 3049794 | 508930 |
| WRLP34 | 71 | 59 | 3050402 | 508930 |
| WRLP35 | 71 | 59 | 3050199 | 508930 |
| WRLP36 | 71 | 59 | 3050198 | 508930 |
| WRLP37 | 71 | 59 | 3050198 | 508930 |
| WRLP38 | 71 | 59 | 3050198 | 508930 |
| WRLP39 | 71 | 59 | 3050198 | 508930 |
| WRLP40 | 71 | 64 | 3049205 | 449233 |
| WRLP41 | 71 | 59 | 3050198 | 508930 |
| WRLP42 | 71 | 61 | 3049794 | 508930 |
| WRLP43 | 71 | 61 | 3049794 | 508930 |
| WRLP44 | 71 | 59 | 3050198 | 508930 |
| WRLP45 | 71 | 59 | 3050198 | 508930 |
| WRLP46 | 71 | 59 | 3050192 | 508930 |
| WRLP47 | 71 | 63 | 3049794 | 508930 |
| WRLP48 | 71 | 59 | 3050198 | 508930 |
| WRLP49 | 71 | 59 | 3050198 | 508930 |
| WRLP50 | 71 | 62 | 3049781 | 508930 |
| WRLP51 | 71 | 61 | 3049922 | 508930 |
| WRLP52 | 71 | 59 | 3050198 | 508930 |
| WRLP53 | 71 | 61 | 3049864 | 508930 |
| WRLP54 | 71 | 61 | 3049794 | 508930 |
| WRLP55 | 71 | 59 | 3050198 | 508930 |
| WRLP56 | 71 | 59 | 3050198 | 508930 |
| WRLP57 | 71 | 61 | 3049790 | 508930 |
| WRLP58 | 71 | 59 | 3050198 | 508930 |
| WRLP59 | 71 | 59 | 3050192 | 508930 |
| WRLP60 | 71 | 59 | 3050198 | 508930 |
| WRLP61 | 71 | 61 | 3049864 | 508930 |
| WRLP62 | 71 | 61 | 3049790 | 508930 |
| WRLP63 | 71 | 61 | 3049794 | 508930 |
| WRLP64 | 71 | 61 | 3049794 | 508930 |
| WRLP65 | 71 | 59 | 3049938 | 508930 |
| WRLP66 | 71 | 59 | 3050198 | 508930 |
| WRLP67 | 71 | 61 | 3049794 | 508930 |
| WRLP68 | 71 | 61 | 3049790 | 508930 |
| WRLP69 | 71 | 59 | 3052878 | 508930 |
| WRLP70 | 71 | 42 | 2878023 | 475167 |
| WRLP71 | 71 | 55 | 3047697 | 508930 |
| WRLP73 | 71 | 35 | 2913628 | 558736 |
| WRLP74 | 71 | 28 | 2914396 | 501366 |
| WRLP75 | 71 | 52 | 3047082 | 508930 |
| WRLP76 | 71 | 58 | 3048854 | 508930 |
| WRLP77 | 71 | 50 | 3044695 | 508930 |
| WRLP78 | 71 | 53 | 3048985 | 508930 |
| WRLP79 | 71 | 56 | 3049258 | 508930 |
| WRLP80 | 71 | 33 | 2876072 | 520718 |
| WRLP81 | 106 | 43 | 3014357 | 475227 |
| WRLP82 | 122 | 20 | 2989154 | 541080 |
| WRLP83 | 122 | 20 | 2989142 | 541080 |
| WRLP84 | 122 | 20 | 2989154 | 541080 |
| WRLP85 | 122 | 20 | 2989143 | 541080 |
| WRLP86 | 122 | 20 | 2989142 | 541080 |
| WRLP87 | 122 | 20 | 2989154 | 541080 |
| WRLP88 | 122 | 23 | 2988889 | 541080 |
| WRLP89 | 122 | 20 | 2989143 | 541080 |
| WRLP90 | 122 | 20 | 2989155 | 541080 |
| WRLP91 | 122 | 20 | 2989154 | 541080 |
| WRLP92 | 122 | 20 | 2989148 | 541080 |
| WRLP93 | 122 | 20 | 2989143 | 541080 |
| WRLP96 | 131 | 27 | 2873895 | 510040 |

**Table S2** | NCBI accession numbers used to screen for antimicrobial tolerance, stress, and virulence genes.

| **Gene/gene region** | **Accession no.** |
| --- | --- |
| *tetR, tnpABC* | HF565366.1 |
| *qacH* | HG329628 |
| *qacC* | QXKR01000002.1 |
| *emrE* | CP001602.2 |
| *emrC* | CP038643.1 |
| *bcrABC* | JX023276.1 |
| *cadA1* | L28104 |
| *cadA2* | AADR01000058 |
| LIPI-1, SSI-1, *inlA, inlB, inlC, inlE, inlF, inlG, inlH, inlI, inlJ, inlK, vip, ami* | NC_003210 |
| *inlC2, inlD* | NC_017544 |
| LIPI-3 | NZ_JABXLV000000000.1 |
| LIPI-4 | FM242711.1 |
